# Discovery of stroke-related blood biomarkers from gene expression network models

**DOI:** 10.1186/s12920-019-0566-8

**Published:** 2019-08-07

**Authors:** Konstantinos Theofilatos, Aigli Korfiati, Seferina Mavroudi, Matthew C. Cowperthwaite, Max Shpak

**Affiliations:** 1 InSyBio: Intelligent Systems Biology, Austin, TX USA; 2 Technological Educational Institute of Western Greece, Patra, Greece; 3 St. David’s Medical Center, Austin, TX USA; 4 Center for Systems and Synthetic Biology, University of Texas at Austin, Austin, TX USA; 5 Fresh Pond Research Institute, Cambridge, MA USA

**Keywords:** Stroke, Gene expression, Gene networks, Biomarkers

## Abstract

**Background:**

Identifying molecular biomarkers characteristic of ischemic stroke has the potential to aid in distinguishing stroke cases from stroke mimicking symptoms, as well as advancing the understanding of the physiological changes that underlie the body’s response to stroke. This study uses machine learning-based analysis of gene co-expression to identify transcription patterns characteristic of patients with acute ischemic stroke.

**Methods:**

Mutual information values for the expression levels among 13,243 quantified transcripts were computed for blood samples from 82 stroke patients and 68 controls to construct a co-expression network of genes (separately) for stroke and control samples. Page rank centrality scores were computed for every gene; a gene’s significance in the network was assessed according to the differences in their network’s pagerank centrality between stroke and control expression patterns. A hybrid genetic algorithm – support vector machine learning tool was used to classify samples based on gene centrality in order to identify an optimal set of predictor genes for stroke while minimizing the number of genes in the model.

**Results:**

A predictive model with 89.6% accuracy was identified using 6 network-central and differentially expressed genes (*ID3, MBTPS1, NOG, SFXN2, BMX, SLC22A1*), characterized by large differences in association network connectivity between stroke and control samples. In contrast, classification models based solely on individual genes identified by significant fold-changes in expression level provided lower predictive accuracies: < 71% for any single gene, and even models with larger (10–25) numbers of gene transcript biomarkers gave lower predictive accuracies (≤ 82%) than the 6 network-based gene signature classification. miRNA:mRNA target prediction computational analysis revealed 8 differentially expressed micro-RNAs (miRNAs) that are significantly associated with at least 2 of the 6 network-central genes.

**Conclusions:**

Network-based models have the potential to identify a more statistically robust pattern of gene expression typical of acute ischemic stroke and to generate hypotheses about possible interactions among functionally relevant genes, leading to the identification of more informative biomarkers.

**Electronic supplementary material:**

The online version of this article (10.1186/s12920-019-0566-8) contains supplementary material, which is available to authorized users.

## Background

The identification of biomarkers characteristic of acute ischemic stroke (AIS) is important both from the standpoint of basic research and for clinical practice. Distinguishing actual instances of AIS from stroke mimics is critical for the triage process in emergency medicine. In addition to providing a method for corroborating diagnosis of AIS, stroke biomarkers have the potential to serve as predictors of stroke severity and clinical outcomes when the abundance of a particular gene product has a significant association with patient outcome measures. Biomarker discovery can also provide additional insight into the basic physiology and molecular biology surrounding AIS and similar infarctions, including apoptosis of brain cells, by identifying genes in key regulatory pathways.

Several recent studies have identified molecular AIS biomarkers from blood samples. Among the biomarkers considered are the standard metabolites assayed in hospital labs, mRNA expression array, RNASeq data, miRNA data, and mass spectroscopy proteomic data [1–3]. One of the molecular markers most strongly associated with AIS is the tetracicopeptide repeat protein *TTC7B*, which is responsible for localizing the P14K kinase in the plasma membrane. Mutations in *TTC7B* have been found to be associated with stroke risk, and the gene appears to be downregulated in stroke patients in comparison to control samples [4]. Similarly, miRNA panel assays found higher blood *let-73-5p* levels associated with downregulation of *CASP3* and *NLK* in AIS patients compared to controls [5].

The conventional approach taken in most of these studies is to compare gene expression levels (or metabolite densities) between an experimental and control group of samples, which in this context means samples taken from stroke patients vs. non-stroke patients. Gene expression comparisons are made by computing the log fold change (FC) in relative transcript densities between stroke and non-stroke in order to determine the extent to which a gene’s level of expression is up or down-regulated between (for instance) stroke vs. non-stroke cases.

While simple and efficient, such approaches suffer from several drawbacks. Among these is the fact that even with *p*-value corrections for multiple comparisons, the number of candidate significant genes remains too high to be of practical value as biomarkers. Furthermore, the genes identified from FC often have no functional relationship with one another, nor do comparisons of gene expression levels identify associations among genes in common pathways. As a result, even if some transcriptomic analyses of stroke patients seem promising and were validated with additional qPCR experiments (e.g. [4], which identified *TTC7B*), the results were not reproducible in further studies and thus their predictive performance for the diagnosis of stroke is limited.

Due to the limitations in the analyses of expression data that are based solely on FC differences, a number of recent studies, e.g. [6–8] have applied machine learning algorithms such as support vector machines, discriminant analysis, and k-nearest neighbour (KNN) clustering to identify more statistically robust set of genetic predictors that can consistently distinguish stroke from non-stroke cases. The present study uses such methods in combination with gene expression network models as a novel approach to stroke biomarker discovery. Analyses of gene expression data make increasing use of network-based approaches that identify covarying transcription patterns among genes [9–12]. Several algorithms have been used to construct gene networks from co-expression data: usually graph edges between pairs of genes are identified from their expression covariances or mutual Shannon-Weaver information measures [12]. The significance of a gene within the network can be quantified in terms of its topological relationship to the other genes – potentially indicating that a gene plays a key regulatory role in an expression pathway. Such network significance metrics are functions of the degree (number of edges) of each node and the weighted degrees of some neighborhood set. Specifically, the “centrality” of a node, computed from its own degree and a weighted count of its neighbors’ (defined up to some Hamming distance) determines its topological importance in a network. A number of network measures can be used to quantify the functional importance of genes in an interaction network, including eigenvector centrality and Page centrality [13, 14].

There has been limited application of network centrality measures to the identification ofg stroke biomarkers. One such study [15] used network models and PageRank centrality to identify significant miRNA-mRNA interactions in animal models of AIS and validated the significant mRNAs by fold change comparisons of human gene expression levels in stroke vs. control patients. In this study, we analyse gene expression data from the blood samples of AIS patients and control groups to construct gene expression network models. The functional importance of genes will be determined by their network centrality, the differences in gene centrality between stroke and control samples will be used to identify stroke biomarkers using machine learning approaches. The efficacy of this network-based method will be compared to the traditional approaches based on the magnitude of difference (FC) in transcript abundance between stroke and control samples.

## Methods

### Datasets

The Gene Expression Omnibus (GEO) was queried to obtain expression profile data in blood samples taken from stroke patients. The transcriptomics data for both AIS and control (non-stroke) samples were obtained from published stroke gene expression studies [4, 16, 17]. All of these data were from microarray experiments (Affymetrix whole-genome expression arrays U133 2.0) on peripheral blood samples from stroke patients and from control non-stroke patients.

### Data integration

Analysis of the pooled data requires integration of the different expression array datasets from [4, 16, 17] into a single expression matrix with a consistent scaling of the expression levels. Dataset [4] consists of 20 stroke and control peripheral blood mononuclear cell (PBMC) samples, [16] has 39 stroke and 25 control whole blood samples, while [17] also has whole blood for 23 stroke and control samples. Because the three datasets were generated via different instrumentation and experimental setup, they were separately and differently normalized in their initial format. Therefore, we also initially re-normalized each data set separately using robust multi-array averaging (RMA [18]) and log transformation of the data. We note that the stroke samples in [16] were evaluated at three time points (within 3 h, 5, and 24 h of the stroke event); only the 3 h time point data were used for this study.

Additionally, pooling data from different studies meant combining expression arrays derived from whole blood samples vs. those from PBMC. Because PBMC excludes non-nucleate cells, one may expect somewhat different mRNA profiles with respect to whole blood due to differences in cell/tissue type. Consequently, it was necessary to determine whether pooling PBMC and whole blood data may introduce artifacts due to mRNA profile differences between the different types of blood samples. This was achieved by comparing the FC per-gene in the whole blood only datasets to the pooled blood/PBMC data using Spearman rank-based correlation analyses.

The data sets were merged by performing a second layer of joint normalization similar to the standard approaches used for qPCR data [19]. Initially, we selected 8 commonly used housekeeping genes (*ACTB, B2M, HMBS, HPRT1, RPL13A, SDHA, TBA, YWHAZ*) that were expressed at comparable mean levels in both treatment (stroke) and control groups and followed the procedures outlined in [19] to identify the minimal subset of genes that show the most among-experiment variability to use as normalizers. Because all of these genes showed relatively high FC across samples (Log_2_FC prior to median per-sample normalization was > 0.3 for all the examined housekeeping genes), normalization was performed based on median per-sample expression level rather than rescaling with respect to expression levels of housekeeping genes.

The pooled data were filtered so that only genes with less than 10% missing expression values would be retained for further analysis. For the remaining missing values, the KNN-Impute method [20] was applied to properly impute the missing data with K = 20. For the final stage of quality control, outliers were identified with a method based on principal components analysis (PCA) – retaining the principal components that accounted for 90% of covariation, and then applying the local outlier factor (LOF) approach [21] to cluster samples and detect outliers as the unclustered samples. This analysis indicated that less than 5% of the data were marked as outliers, thereby passing a predefined threshold of fewer than 10% outliers for a dataset to be considered valid for further analysis.

### Analysis of differential expression

Analyses of differential expression (fold-change in gene expression levels between stroke and non-stroke samples) were performed using InSyBio’s implementation of limma (linear model for microarray) using the Ebayes algorithm [22] to evaluate linear model fit considering the sample type (PBMC/whole blood) as a covariate. Moderated t-statistics (t-scores where the standard errors are reduced to a common value across probes) were used to determine the *p*-values associated with the log fold-change (log FC), i.e. log_2_[X_S_/X_C_], where X_S_ and X_C_, are, respectively, the mean gene expression levels in the stroke and control samples. The Benjamini-Hochberg false discovery rate (FDR) correction [23] was performed to adjust the p-values for multiple comparison. Statistically significant log FC values were defined as those with FDR-adjusted *p* < 0.05 of the moderated t-statistic.

Differences in instrumentation across samples were considered a potential source of bias, and was removed by specifying this variation as a covariate in the Ebayes algorithm to account for heterogeneity between the combined expression arrays. Genes with statistically significant log FC values between stroke and control samples are retained as input for prediction models. The prediction models developed from this gene set were used for comparison with prediction models using gene sets derived from the network-based methods of identifying significant genes, as outlined below.

### Network-based biomarker characterization

In order to identify biomarkers that are both statistically and potentially functionally significant, the InSyBio Bionets network-based approach was applied to the expression data. The network analyses leverage mutual covariance in expression levels to characterize statistical association of transcription patterns among genes. Following the mutual information-based approach described in [24], a correlation network was constructed for both control and stroke sample expression data. For every gene pair, the mutual information value *I* between the expression levels of two genes *x*,*y* is computed, where for the probabilities (frequencies) of observed states *i,j* ∈ *x,y* and joint frequencies *p*_ij_

$$ {I}_{x,y}=\sum \limits_{i\in x}\sum \limits_{j\in y}{p}_{i,i}\mathit{\log}\left[\frac{p_{ij}}{p_i{p}_j}\right] $$

A first cut-off value of *I* < 0.2 is used to eliminate uncorrelated data. The statistical significance of covariance in expression between the remaining gene pairs was determined based on 95% confidence intervals in a bivariate normal distribution, with the final significance threshold that was used for most gene pairs typically corresponded to mutual information values of *I* = 0.7–1.0. As outlined in [25], weighted edges are assigned to pairs of nodes with significant mutual information.

The centrality of each node (gene) in the graph was determined using the PageRank algorithm [13, 14] – a modified eigenvector centrality algorithm best-known for its use by Google to identify the most relevant matches to search query terms. Centrality scores were assigned to genes in both the stroke and control data sets. The mean log_2_ FC in centrality scores between stroke and control were compared using t-tests (following Spiro-Wilcoxon tests for normality) where statistically significant nodes were defined as those with FDR-adjusted *p* < 0.05. We refer to these as network significant genes, to distinguish this set of genes from those identified by expression level FC significance.

The Gene Ontology (GO) resource [26] and DAVID [27, 28] annotation tools were used to characterize the functional roles and other shared features of centrally significant genes using enrichment analysis of the gene set with respect to functional roles and pathways. In addition, the Hamming distance d = 1 neighbor set of each network-central gene was compared to the set of genes with known or predicted interactions and functional associations with the network-central genes according to the STRING database tool [29]. Associations of genes in the neighborhood sets with hereditary diseases was assessed using the DisGeNet [30] tool, which assigns an association score to genes whose mutational variants are linked to known diseases in the biomedical literature.

### Characterization of predictive accuracy

The set of genes with significantly different network centrality values was used to develop machine learning models that optimize the predictive accuracy of stroke vs. non-stroke classification using a minimal number of genes. Generating a manageable and robust model for stroke prediction/classification requires optimization with respect to two criteria: the first is the predictive accuracy, i.e. the frequency with which samples are correctly classified as coming from AIS vs. control based on gene expression), and second, simultaneously attempting to minimize the number of genes that are used to generate the predictive model. This algorithm was applied to both the set of network-significant genes and the FC-significant genes in order to compare the relative predictive value of genes identified from networks vs. individual expression levels.

In the optimization process, a multi-objective genetic algorithm (GA) is used to identify the optimal feature set input from a population of float vector solutions (subset of predictor genes and model parameters). The float vector is initialized with a small number of genes, and in each subsequent generation the feature set is added to or subtracted from sequentially via mutation and recombination operators. Replication of a feature set proportional to the fitness of a predictive model. In the multi-objective optimization technique used for this task the overall fitness is calculated using a combination of the following independent fitness functions:

- Fitness Function 1: 1/(1 + Νumber of selected features)
- Fitness Function 2: Classification Accuracy
- Fitness Function 3: Geometric Mean of Sensitivity and Specificity
- Fitness Function 4: Number of Samples in Training Set/Number of Support Vectors of the trained Support Vector Machine Problem

The fitness functions 1 and 4 were used to promote solutions which lead to the simplest, most general possible models. The other fitness functions were used to achieve accurate classification performance, dealing effectively with the imbalanced nature of the dataset. Specifically, a weighted sum of the independent fitness functions was used to calculate the overall fitness of a solution using the following weights: Fitness function 1: 1, Fitness function 2: 5, Fitness Function 3: 5, Fitness Function 4: 5. These weights were selected in order to provide the same (high) emphasis in classification metrics, while considering that the simplicity of the model is of less importance for these problems.

A model’s efficacy was assessed by computing its Predictive Accuracy = (TP + TN)/(TP + TN + FP + FN) with TP: True Positives, TN: True Negatives, FP: False positives, FN: False Negatives. The accuracy of each model was calculated via 5-fold cross-validation of the dataset. For every iteration, 80% of the data (both stroke and control) were used as a training set while the frequency at which the remaining 20% are correctly classified defined the predictive accuracy.

### Differential expression of micro-RNAs

Recent studies have indicated that several miRNAs are associated with stroke-related cellular mechanisms [31, 32]. In order to identify miRNAs that may be acting as regulators of genes that are stroke-predictive, we utilized the InSyBio ncRNAseq tool [33] to identify all miRNAs that can potentially target the six transcripts of interest. For this process we used the default threshold (0.3) as suggested by [34] for the predicted confidence score for each miRNA:mRNA examined found 907 miRNAs. This predicted confidence score is estimated from an epsilon-SVR regression model using 124 sequential, thermodynamic and structural features of all potential miRNA:mRNA target sites and initially trained with a balanced positive and negative miRNA:mRNA pairs. However, not all these microRNAs are expressed across multiple tissue types, therefore, most of them are not identifiable in blood samples while others are not differentiated in stroke patients. These 907 miRNAs were further filtered using the high-throughput transcriptomics data from [35].

Significant differential expression in miRNA between stroke and controls was analysed using this data. The analyses were similar to the workflow for mRNA described in subsection C above, i.e. log FC was computed for miRNA densities in stroke vs. control samples, and statistically significant magnitudes of FC expression level were identified via moderated t-statistics and FDR corrections.

## Results

### Normalization and processed dataset

**Fig. 1:**
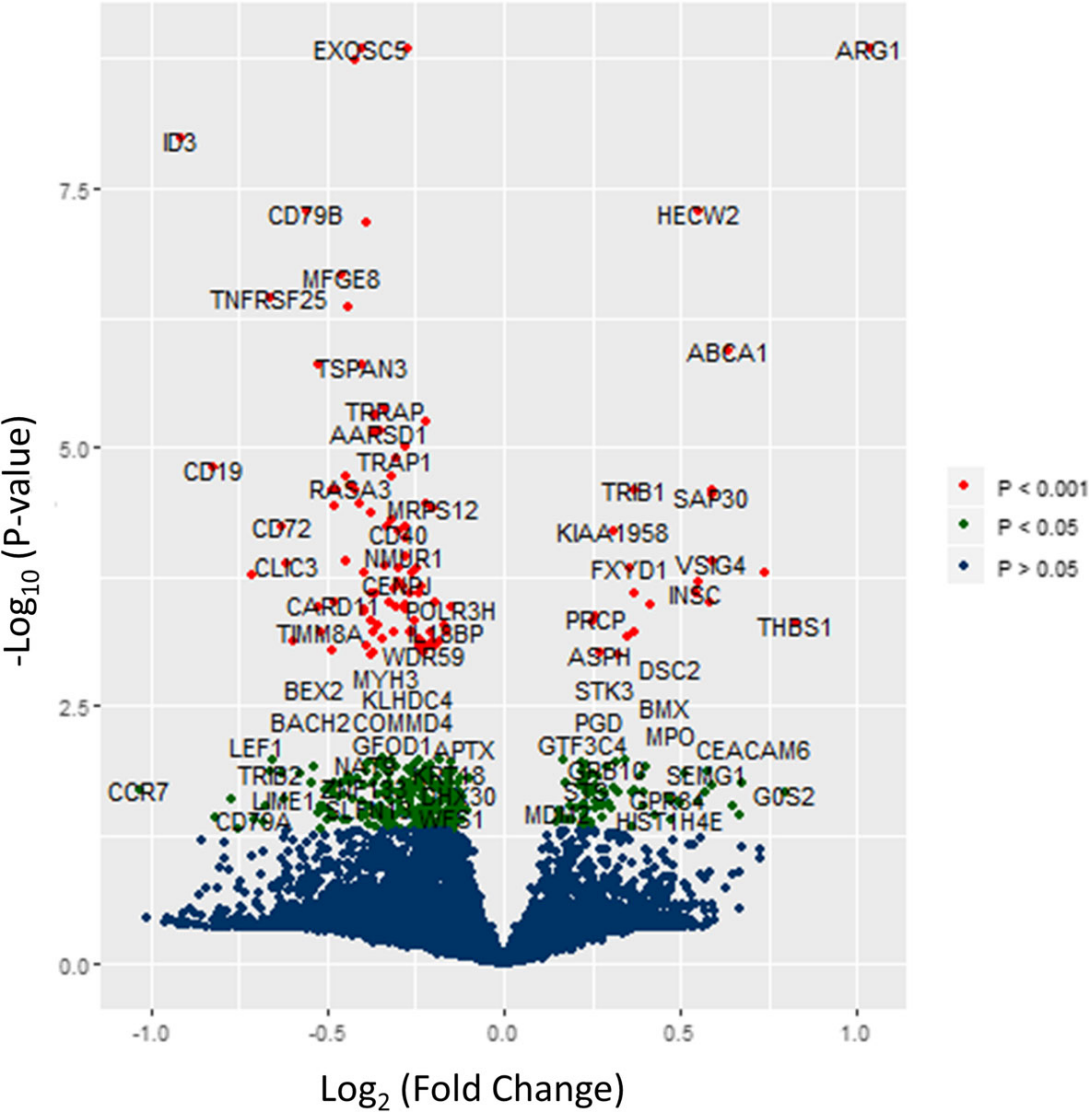
Volcano Plot of Differential Expression Analysis. The genes with the largest and most statistically significant absolute fold-change between treatment and control are those at the upper left and right corners

Following normalization, imputing of missing data, and outlier removal, the total merged dataset consists of 137 samples (82 stroke patients and 55 control subjects), with a total of 13,243 quantified transcripts. The validity of merging PBMC with whole blood datasets was confirmed by analyses of differential expression in the combined vs. whole blood-only samples. Additional file 1: shows a volcano plot of FC vs. *p*-value for the blood only data (compare to Fig. 1 for the pooled data), while the second figure in the supplement shows a scatterplot of FC between blood-only and pooled expression arrays. A Spearman rank-based correlation value of 0.99 and p < < 0.001 confirms the consistency of blood-only and pooled data doesn’t introduce artifacts associated with disparate gene expression profiles. Pooling PBMC and whole blood samples does slightly increase the variance and range in gene expression, which accounts for the fact that 584 significantly differentially expressed genes were found in the whole blood samples vs. 557 in the combined data set.

### Differential expression analysis

**Fig. 2:**
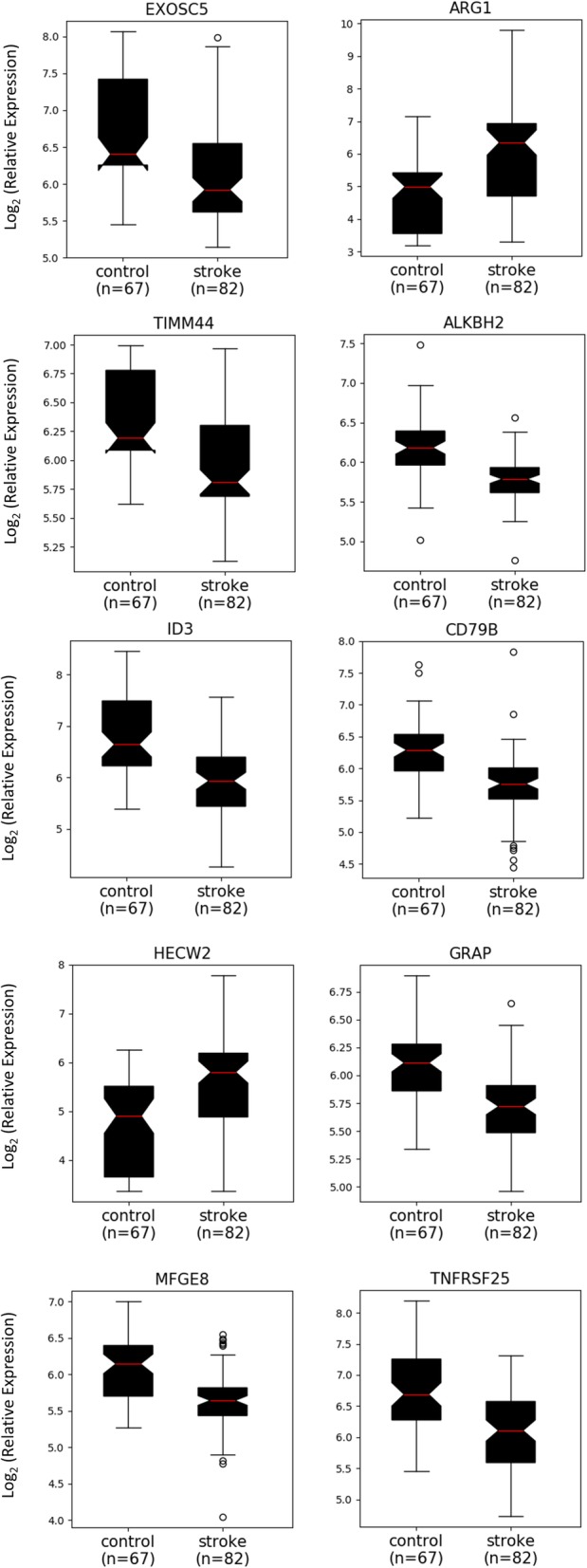
Boxplots showing the range in gene expression for the 10 most statistically significant differentially expressed genes, illustrating the extent of FC and the separation of median expression levels between stroke and control samples

A total of 557 genes are significantly differentially expressed between stroke and non-stroke samples (see Additional file 2: for a complete list). The distribution of log FC and *p*-values for the entire set of genes is shown as a volcano plot in Fig. 1, and Table 1 summarizes the log FC data and statistics for the 10 genes with the most statistically significant (smallest FDR-adjusted p-value based on t-statistics) differential expression between stroke and control samples. The median values in expression level between the stroke and control samples are strongly separated for these 10 genes, as shown in the Fig. 2 boxplot panels.

**Table 1:** Differential gene expression of the 10 genes with the most statistically significant (smallest FDR-adjusted *p*-values) associated with the fold change between stroke and control samples

Gene Symbol	Log FC	Average Overall Expression in the Dataset	t	*p*-value	Benjamini-Hochberg Adjusted p-value
*EXOSC5*	−0.39998	6.43394	−8.12935	1.58E-13	1.41E-09
*ARG1*	1.040751	5.385215	8.073018	2.18E-13	1.41E-09
*TIMM44*	−0.27118	6.158495	−8.00489	3.21E-13	1.41E-09
*ALKBH2*	−0.41991	5.966395	−7.91172	5.43E-13	1.80E-09
*ID3*	−0.91519	6.336414	−7.55943	3.90E-12	1.03E-08
*CD79B*	−0.5584	5.968423	−7.22571	2.44E-11	5.36E-08
*HECW2*	0.550209	5.12878	7.198337	2.83E-11	5.36E-08
*GRAP*	−0.39057	5.862544	−7.12923	4.12E-11	6.82E-08
*MFGE8*	−0.4621	5.820657	−6.88759	1.51E-10	2.22E-07
*TNFRSF25*	−0.66299	6.376879	−6.77806	2.69E-10	3.57E-07

### Network-based biomarkers

Using the criteria of differences in PageRank centrality values between stroke and normal samples, 47 potentially significant differentially expressed genes were identified. The rank list of genes in the differential expression analysis is summarized in the Additional file 3.

**Fig. 3:**
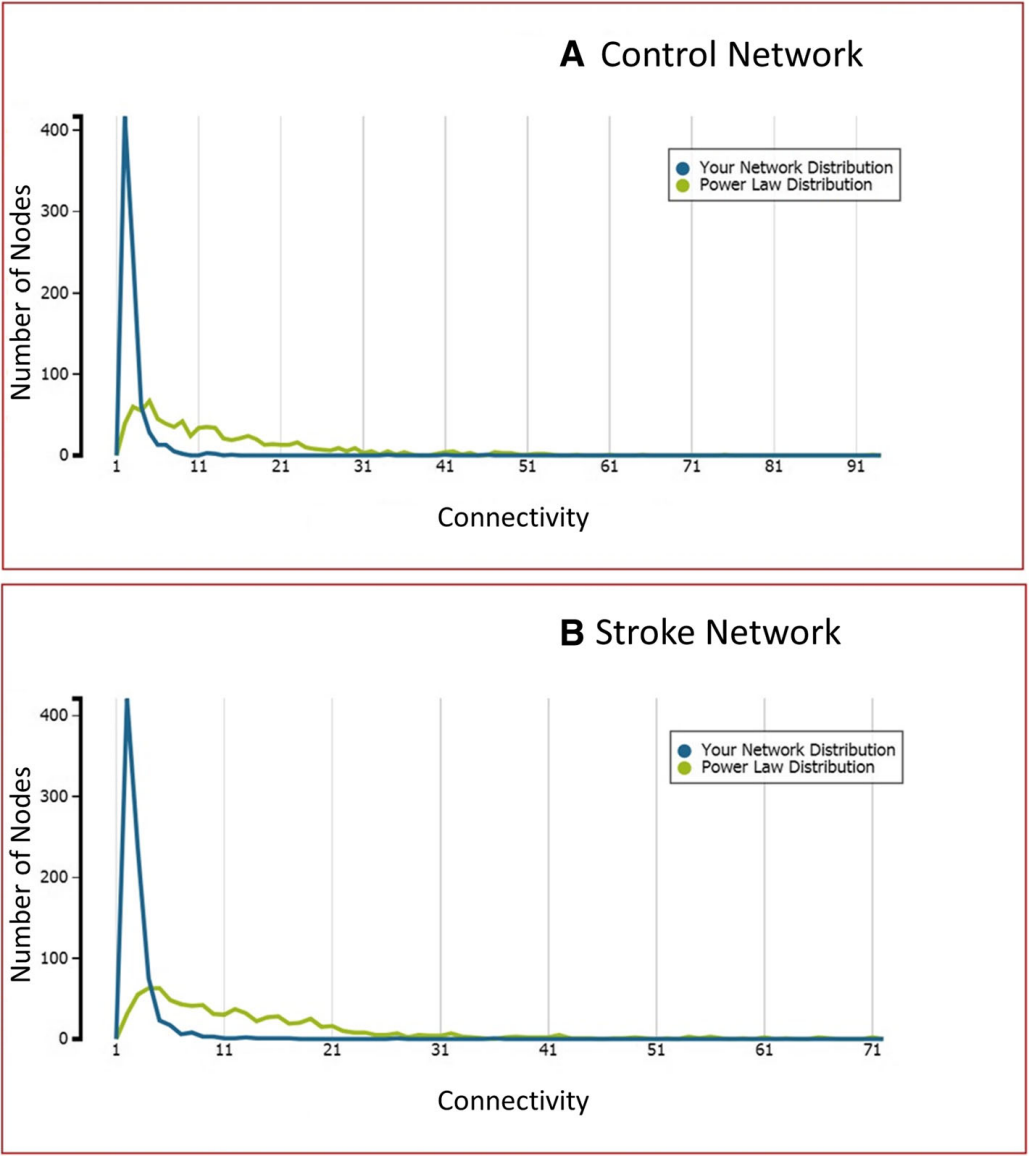
Frequency distribution of node (gene) degrees (edge numbers) for both **a**) the control and **b**) the stroke networks, compared to a model power law distribution of node degree. Blue line depicts the node degrees frequency distribution for the recontrsucted networks while green line depicts the anticipated node degrees frequency distribution based on the power law model

Both the stroke and control networks have high connectivity, with statistically significant edges linking most groups of genes in such a way that there are few disjoint sets and the path distance between randomly selected genes is short. This can be seen from the distribution of node (gene) degrees over the entire gene set is summarized in Fig. 3, which compares connectivity within the stroke and control networks to a power law distribution. Due to a larger number of degree 1–2 nodes in comparison to intermediate or high degree nodes, the fit of the observed node degree distribution in the gene networks to a power law distribution appears poor. However, a Spearman rank correlation analysis comparing predicted to observed degrees give correlation coefficients ρ = 0.596 and 0.612 for stroke and control, respectively, with *p* < 0.001, indicates a statistically significant concordance between the observed distribution and a power law. This pattern is consistent with “small world” phenomena [36], where every node connects to most other nodes through a comparatively short path. This high connectivity indicates at least an indirect statistical interaction between co-expression levels of most genes, especially in the stroke samples. Specifically, the expected number of edges connecting a random gene pair increases logarithmically with the number of genes, i.e. at a less than linear rate.

### Predictive analytics

**Fig. 4:**
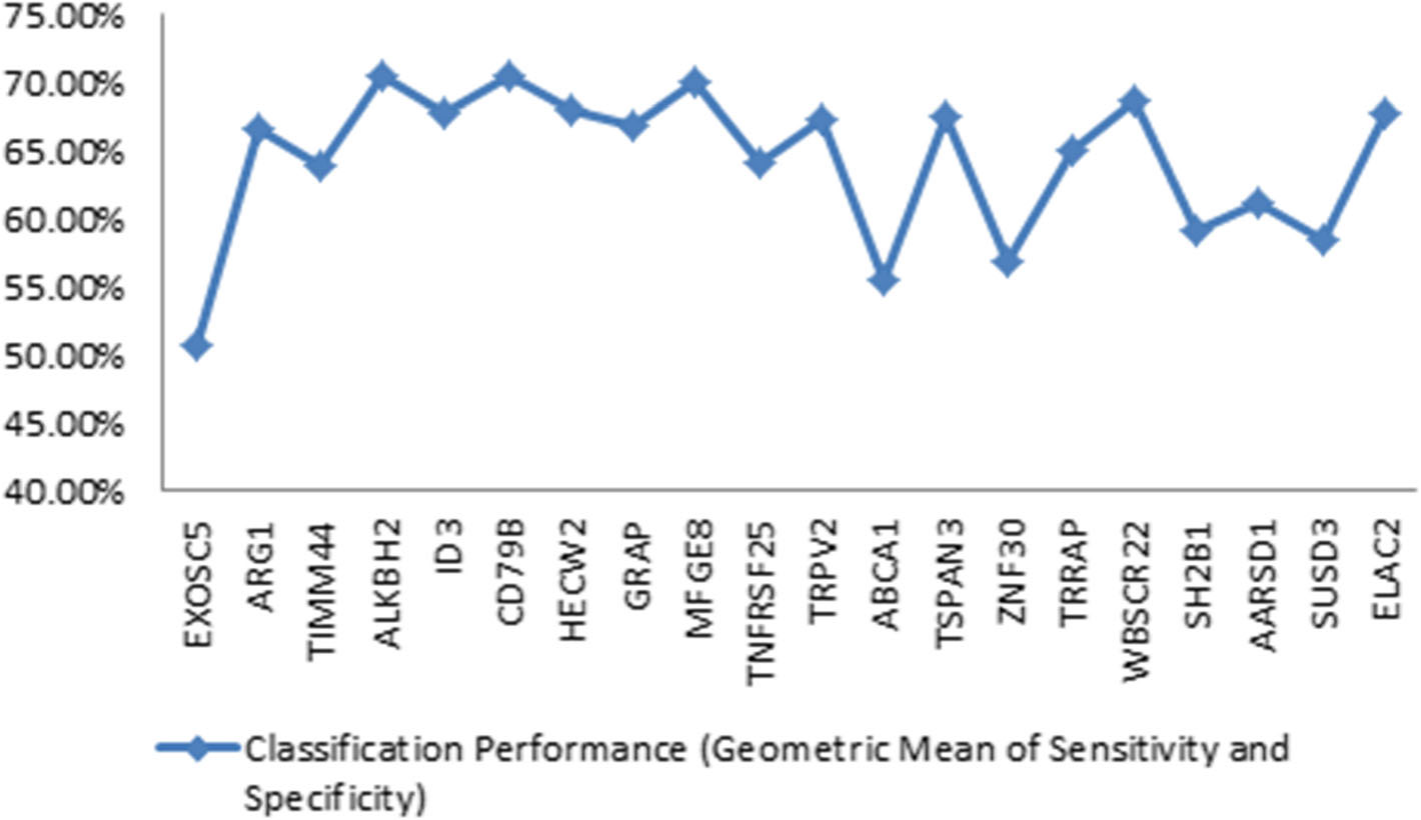
Predictive accuracies of the 10 genes with the strongest differential expression (see Table 1) between stroke and control, illustrating how no gene provides a predictive accuracy individually exceeding 70%

Single-gene expression levels give a maximum predictive accuracy of 70%, this can be seen from the plot of individual predictive accuracy among the 10 most strongly differentially expressed genes in Fig. 4 (with DNA oxidative demethylase *ALKBH2* and lactadherin *MFGE8* giving the highest predictive accuracies of 0.70). Furthermore, when the 10 most highly dysregulated genes are used jointly in machine learning models, the predictive accuracy remains at or below 75.1%. The same is true if a larger set of highly dysregulated genes is selected using the same optimization criteria (objective function) as for selection of the network gene set, i.e. a model with 26 genes selected by the GA-SVM optimization algorithm only increases the predictive accuracy to 81.2% (see Table 2). A model using the 10 genes identified by O’Connell et al. [8] gave predictive accuracy of 82.1% with cross-validation sampling, as shown in Table 2.

**Table 2:** Comparison of gene set and predictive accuracy based on log FC expression level significance vs the network-based model. The first row is for predictive models using single differentially expressed genes; the second is based on the 10 most significant FC values (see Table 1); the third uses the hybrid machine learning algorithm to identify a set of predictor genes from the 557 signficant FC genes; the fourth uses 10 genes identified by O’Connell et al’s predictive model; the last row uses the 6 network-central genes in the prediction model

Method	Number of biomarkers	Predictive Accuracy (results with 5-fold cross validation)
Differential Expression analysis	557	< 71%
Support Vector Machines (default parameters) using the10 most differentially expressed genes as input	10	75.1%
InSyBio predictive analytics approach using differentially expressed gene set	25	81.21%
Gene expression signature from O’Connell et al. 2017	10 (*ANTXR2, STK3, PDK4, CD163, MAL, GRAP, ID3, CTSZ, KIF1B, PLXDC2*)	82.07%
InSyBio predictive analytics using network significant gene set	6 (*ID3, MBTPS1, NOG, SFXN2, BMX, SLC22A1*)	89.57%

Application of feature selection to the significant network-based biomarkers led to the identification of 6 genes whose expression values gave higher joint predictive accuracy than any combination of genes from the differential expression analysis (Table 2). In what follows, we will refer to this set as “network-central predictors,” in reference to the fact that they were identified by significant differences in network centrality between stroke and control expression arrays. As seen in Table 2, expression values from the genes *ID3, MBTPS1, NOG, SFXN2, BMX,* and *SLC22A1* can jointly distinguish stroke from non-stroke samples with a high level of accuracy: 89.6% of samples are correctly classified in cross-validation sampling. As a qualitative comparison, a predictive model based on the 10 genes in [8] had 82.07% accuracy when trained with the dataset of the current study and evaluated with the same cross-validating sampling strategy.

While most of the 6 network central genes also have significantly different expression levels between stroke and control, with the exception of *ID3*, they are not necessarily among the set of most highly dysregulated genes. Indeed, the differences in expression level for *SLC22A11* between stroke and control are not even statistically significant. The mRNAs of *ID3*, *MBTPS1*, *NOG*, and *SFXN2* have lower concentrations (are down-regulated) in the stroke samples (log FC = − 0.915, − 0.356, − 0.752, − 0.301, respectively) while *BMX* and *SLC22A11* are up-regulated (log FC = 0.456, 0.365) in the stroke patients.

**Fig. 5:**
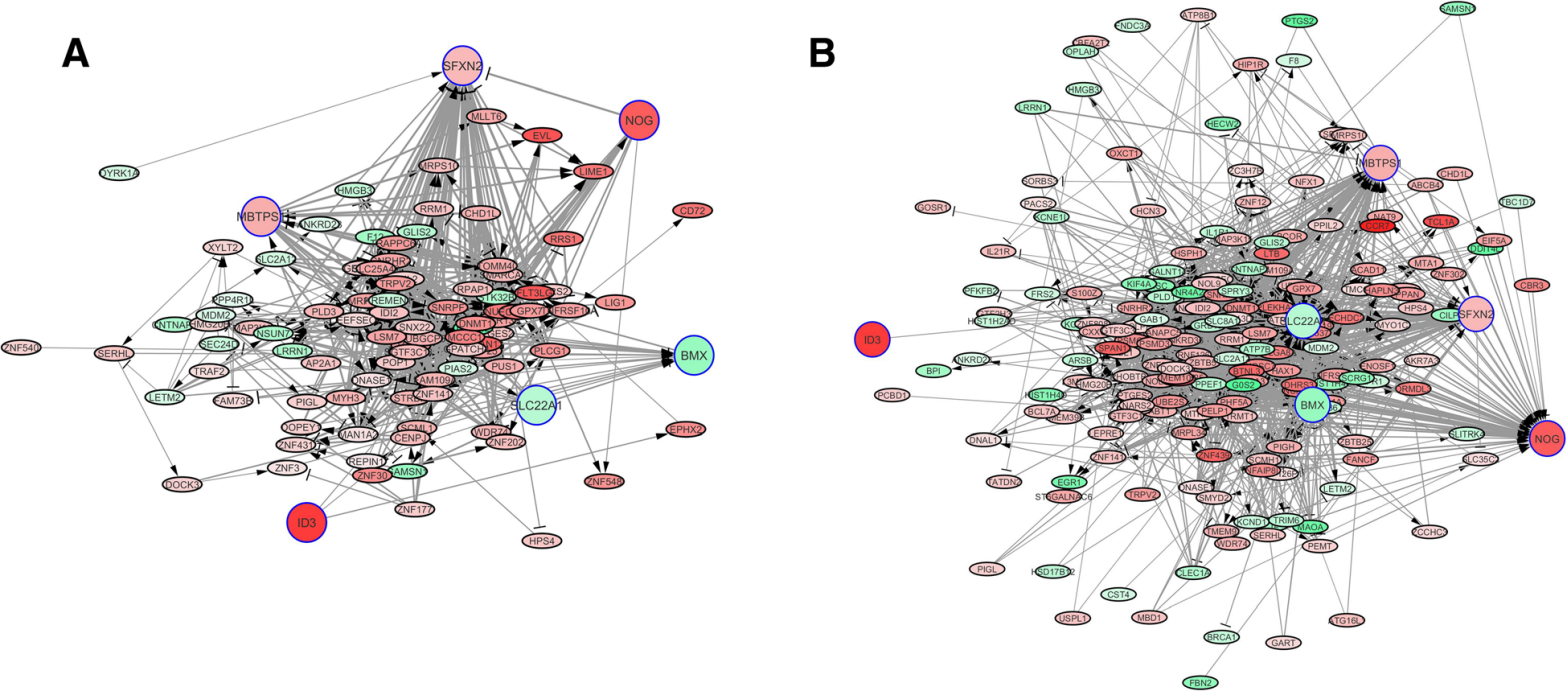
**a** Partial gene network for the 6 network-central predictor genes (ID3, MBTPS1, NOG, SFXN2, BMX, SLC22A1) for network of gene associations from control (non-stroke) samples. Neighborhood sets of Hamming distance d ≤ 2 are shown for each gene, based on statistically significant edges between gene pairs. In order to highlight the strongest associations and improve readability, only those edges corresponding to a mutual information values I ≥ 0.75 are shown. **b** A partial gene network, as in **a**, for the stroke samples

These network-central genes have intersecting sets of Hamming distance 1,2 neighbors. Indeed some gene pairs are within mutual d = 1 neighborhoods (e.g. *SLC22A1* is a d = 1 neighbor to *SFXN2, BMX, NOG*, and *MBTPS1*, and all of the gene pairs except those with *ID3* are within mutual Hamming distance d ≤ 2 of one another). Consequently, none of these neighborhoods are mutually disjoint, so that no more than two significant associations (edges) separate any pair from the 6 genes. This can be seen in Fig. 5a-b, which respectively show the d ≤ 2 neighborhoods of the 6 network-central genes in the control and stroke networks (note that these graphs are somewhat “truncated” for readability by showing only the edges with *I* ≥ 0.75). This high connectivity is consistent with the distribution of node degrees and fit to power law distributions shown in Fig. 3, and with the fact that these genes have physiologically related functions associated with secretion and cell signalling.

However, there are significant differences in connectivity between the stroke and control networks. For example, Fig. 5b shows a single *I* ≥ 0.75 connection for *ID3*, vs. 3 in the control network. If all d = 1 neighbors are considered without the truncation (see Additional file 4), there are actually 7 neighbors in the stroke network and 9 in the control, indicating a small decrease in connectivity – none of the genes in the two networks are shared, which accounts for the statistically significant changes in network centrality. In contrast, the connectivity of the other 5 genes is higher in the stroke vs. control networks: for *MBTPS1*, the control and stroke d = 1 neighborhoods have 20 vs. 49 genes, respectively, for *NOG* 13 vs. 65, for *SFXN2* 42 vs. 62, for *BMX* 22 vs. 61, and for *SLC22A1*, 15 vs. 63 neighbors.

None of the genes in the d = 1 neighborhoods of the network-central gene set are identified as neighbors in STRING db. However, in the case of *SFXNL*, STRING db identifies functionally similar genes to those in the neighborhood set of our networks. Specifically, STRING db lists solute carriers *SLC25A19, 25A1* and heat shock protein *HSPA14* as interacting with *SFXNL*. The d = 1 neighborhoods for *SFXNL* in our analyses include the solute carriers *SLC22A1, SLC41A1* and the heat-shock protein *HSPH1*.

### Functional annotation

The six network-central predictor genes are functionally disparate, however, mosts of them are.involved in secretory and signalling pathways. The inhibitor of DNA binding *ID3* interferes with the binding of helix-loop-helix proteins; the protein noggin (*NOG*) binds and inactivates TGF-beta growth factors; membrane bound transcription factor site-1 protease *MBTPS1* processes proteins through secretory pathways; cytoplasmic tyrosine protein kinase *BMX* is a receptor molecular involved in several transduction pathways, solute carrier *SLCA11* is a voltage-gated transporter, and sideroflexin *SFXN2* regulates cation transport.

Enrichment analyses of d = 1 network neighborhoods of the 6 predictor genes reveal several functional and structural classes of genes associated with changes to network centrality in stroke patients – significant enrichment classes are summarized in Table 3, while Additional file 5: contain the complete DAVID enrichment analysis tables.

**Table 3:** Enrichment by function of gene sets derived from significant FC and from Hamming distance d ≤ 1 neighborhoods of the network-based predictor genes ID3, MBTPS1, NOG, SFXN2, BMX, SLCA11 in the stroke networks. The last column shows the number of genes in the cluster, the associated enrichment odds ratio, the raw/Benjamini-Hochberg FDR-adjusted p-value for the OR are p/p*

Gene Set	Number of Genes	Functional Classes	Subset, OR, p/p*
Genes with significantly different centrality values between stroke and control networks	47	nucleoplasm	34, 2.18, 0.005/0.344
*ID3* d ≤ 1 neighbors in stroke network	7	Negative regulation of apoptotic process in bone marrow	2, 1049.5, 0.002/0.198
		Transcription factor binding	3839.6, 0.006/0.274
		Cytoplasm	6, 22.29, 0.024/0.394
*MBTPS1* d ≤ 1 neighbors in stroke network	53	Zinc finger	3,89.23,4.8 × 10^−4^/0.077
		Ligase	5,5.60,0.011/0.978
		Transmembrane helix	23,1.581,0.014,0.427
		Lumenal topological domain	5, 4.21,0.029/0.909
*NOG* d ≤ 1 neighbors in stroke network	70	Transmembrane transport	3,39.57,0.002/0.727
		Endoplasmic reticulum lumen	5,6.98,0.005,0.437
		Regulation of microvillus assembly	2, 100.25, 0.020/0.994
		Arachinodic acid metabolism	3,11.94,0.024/0.795
		Poly(A) RNA binding	10,2.27,0.028/0.991
*SFXN2* d ≤ 1 neighbors in stroke network	65	Phosphoprotein	39, 1.52, 0.001/0.124
		Mitochondrial	13,2.83,0.002/0.156
		Acetylation	19,1.78,0.002/0.136
*BMX* d ≤ 1 neighbors in stroke network	62	Nucleotide-binding	14,2.60,0.002/0.257
		Nuclear localization signal	6,5.64,0.004/0.664
		ATP-binding	11,2.63,0.004/0.664
		Phosphoprotein	
*SLC22A1* d ≤ 1 neighbors in stroke network	65	Mitochondrion	11,3.07,0.003/0.344
		Protein binding	42,1.28,0.017/0.929
		Nucleolus	8,2.74,0.024/0.914
		DNA catabolic process	2,66.63,0.020/1.0
		ATP-binding	10, 2.24,0.030,0.815

For the 47 genes with significant differences in their network centrality between stroke and non-stroke, there is significant enrichment of nucleoplasm proteins (number of genes n_g_ = 15, OR = 2.18). There are high odds ratios associated with enrichment of other functional and structural classes of genes, e.g. U-box domain genes (n_g_ = 2, OR = 121.96), but these are not statistically significant.

The relatively small neighborhood of *ID3* (7 genes with shared edges + *ID3* itself) contain 2 genes involved in repression of apoptosis in bone marrow (*LEF1, FLT3LG*, with OR = 25) as well as enrichment for transcription factor binding genes (*LEF1, ID3, TRIB2*, OR = 37.5). For *MBTPS1*, the strongest enrichments are seen for zinc finger proteins (n_g_ = 23, OR = 89.23) and for ligases (5, n_g_ = 5.60). The *NOG* neighborhood is enriched for 5 genes (OR = 6.89) in the endoplasmic reticulum, 2 genes of the microvillus assembly (OR = 100.25, and 10 genes whose proteins bind poly(A) RNA (OR = 2.27). Of the 65 genes in the *SFXN2* d = 1 neighborhood, 39 are phosphoprotein (OR = 1.52), 13 are mitochondrial (OR = 2.83), and 19 are involved in acetylation (OR = 1.78). In the degree 1 neighborhood of *BMX*, the strongest enrichments are for nucleotide and ATP-binding (14 and 11 genes with OR = 2.60, 2.63, respectively) and 6 genes for nuclear localization (OR = 5.64). *SLC22A1*, being within d = 1 of *SFXN2*, also has a neighborhood enriched in mitochondrial genes (n_g_ = 11 of the 65 neighbors + *SLC22A1*, OR = 3.07), as well as nucleolar (n_g_ = 8, OR = 2.74 and ATP-binding (n_g_ = 10, OR = 2.24).

**Fig. 6:**
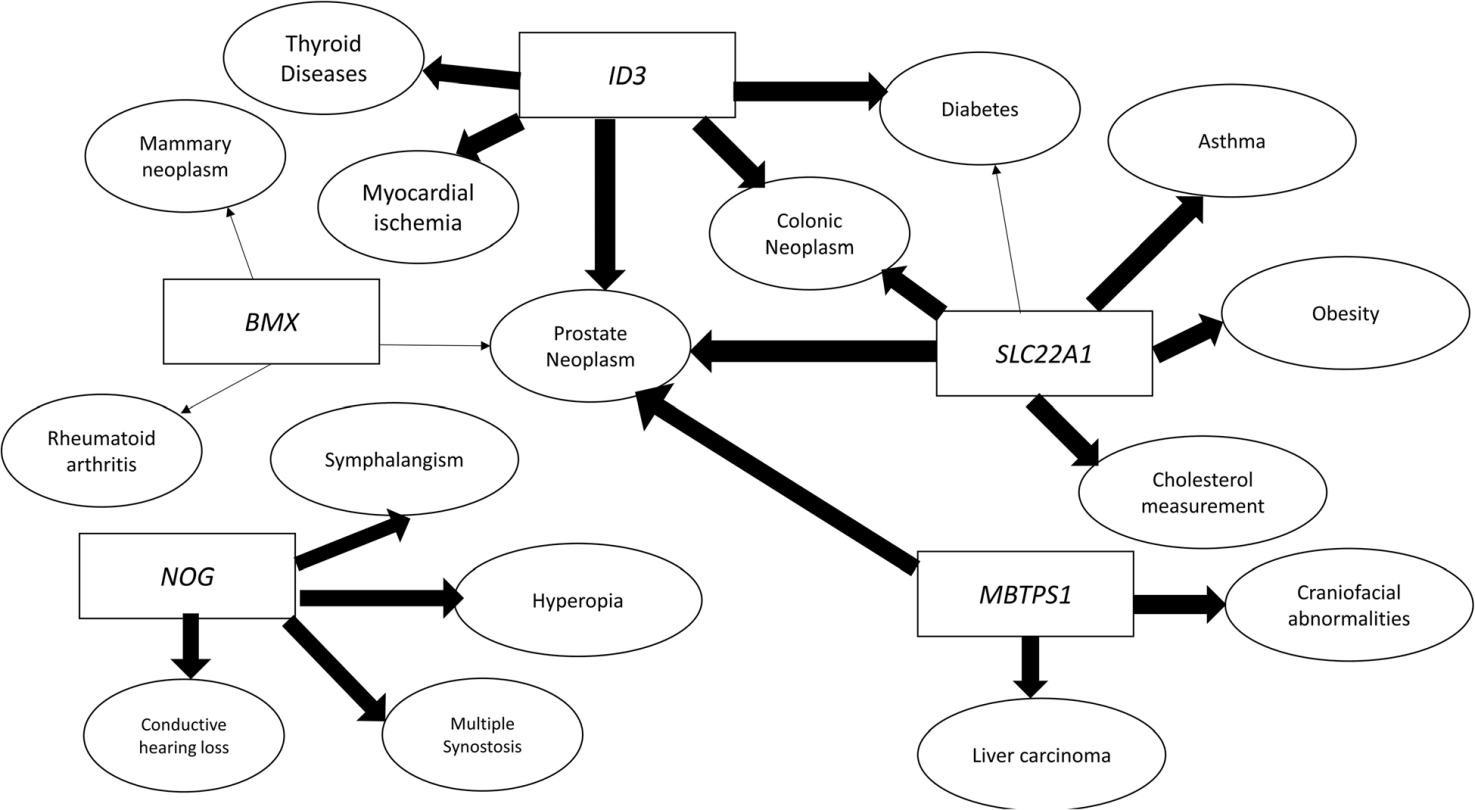
Association of 5network-central predictor genes (ID3, MBTPS1, NOG, BMX, SLC22A1) with known diseases according to the DisGeNet database using the default threshold of 0.001 (no such associations were identified for SFXN2)

A DisGeNet [28] search of the 6 network-central genes identified several diseases associated with their mutational variants or dysregulation in the literature. Figure 6 summarizes the diseases with a threshold association score > 0.001 for each gene (see Additional file 6: for a complete list); only *SFXN2* had not been previously significantly associated with any disease in the database While the other genes have not been previously linked to AIS, they have been linked with diseases and comorbidities such as diabetes (*ID3, SLC22A1*), obesity (*SLC22A1*), and myocardial ischemia (*ID3*).

### Stroke related miRNAs

A set of 115 miRNAs were found to be significantly differentially expressed between stroke and controls. Following the preliminary filtering based on confidence scores, this initial set of miRNAs was reduced to 27 (Table 4, see Additional file 7: for a complete list of associations between miRNA and the network-central 6-gene set). Among this set of miRNAs, eight (hsa-miR-1181, hsa -miR-1207-3p, hsa -miR-1246, hsa -miR-3180, hsa -miR-3960, hsa -miR-4436a, hsa -miR-517a-3p, hsa -miR-517a-3p) were determined to target two or more transcripts from the set of 6 network-central predictor genes, with the exception of *SFXN2*. The increased correlation between the stroke related miRNAs identifiable in blood and the set of predictor genes provides an additional validation of the statistical and potentially functional significance of these genes as stroke biomarkers.

**Table 4:** MiRNAs targeting the 6 significant genes (confidence score > 0.3) and their expression profile in blood samples of ischemic stroke patients from [34]

MiRNA	Predicted Targets and Confidence Score	Expression in Blood samples of Ischemic Stroke Patients
hsa-miR-1181	*ID3* (1.223), *MBTPS1* (0.454), *SLC22A1*(0.302)	Downregulated
hsa-miR-1207-3p	*BMX* (0.896), *NOG*(0.687), *MBTPS1* (0.57), *SLC22A1* (0.404)	Downregulated
hsa-miR-1229-3p	*ID3* (0.555)	Downregulated
hsa-miR-1246	*NOG* (0.359), *MBTPS1*(0.331)	Upregulated
hsa-miR-1262	*NOG* (0.305)	Downregulated
hsa-miR-138-2-3p	*NOG* (0.45)	Downregulated
hsa-miR-1909-5p	*NOG* (0.445)	Downregulated
hsa-miR-199a-5p	*ID3* (0.338)	Upregulated
hsa-miR-29c-5p	*MBTPS1* (0.315)	Downregulated
hsa-miR-3129-5p	*ID3* (0.395)	Downregulated
hsa-miR-3180	*MBTPS1* (0.782), *NOG* (0.782), *ID3* (0.587), *SLC22A1* (0.556)	Downregulated
hsa-miR-3180-3p	*NOG* (0.539)	Downregulated
hsa-miR-3612	*BMX* (0.554)	Downregulated
hsa-miR-3620-3p	*ID3* (0.786)	Downregulated
hsa-miR-3657	*MBTPS1* (0.481)	Downregulated
hsa-miR-371a-3p	*NOG* (0.703)	Downregulated
hsa-miR-3960	*MBTPS1* (1.179), *NOG* (0.868), *ID3* (0.532), *SLC22A1* (0.439)	Upregulated
hsa-miR-4259	*NOG* (0.349)	Downregulated
hsa-miR-4436a	*MBTPS1* (0.601), *SLC22A1* (0.339), *NOG* (0.314)	Downregulated
hsa-miR-4725-5p	*ID3* (0.405)	Upregulated
hsa-miR-517a-3p	*ID3* (0.79), *SLC22A1* (0.343)	Downregulated
hsa-miR-517b-3p	*ID3* (0.79), *SLC22A1* (0.343)	Downregulated
hsa-miR-520a-3p	*NOG* (0.348)	Downregulated
hsa-miR-532-5p	*MBTPS1* (0.323)	Downregulated
hsa-miR-548n	*NOG* (0.362)	Downregulated
hsa-miR-551b-3p	*MBTPS1* (0.302)	Downregulated
hsa-miR-5587-3p	*ID3* (0.615)	Downregulated
hsa-miR-5588-5p	*ID3* (0.392)	Downregulated
hsa-miR-607	*ID3* (0.388)	Downregulated
hsa-miR-615-5p	*NOG* (0.39)	Downregulated

## Discussion

Previous studies have used gene sets identified from FC significance to achieve similar predictive accuracies stroke vs. controls samples via machine learning models. For example, [6] identified a set of 29 genes that provided 93.5% sensitivity and 89.5% specificity in distinguishing control vs. stroke, while [7, 8] used expression levels from a panel of 10 genes to achieve 95.6% predictive accuracy. None of the 29 genes in [6] are in our set of 6 network central genes, while of the 10 in [7, 8], *ID3* is the only shared gene.

The predictive accuracies in these studies vs. our results are not directly comparable, insofar as we use a different (merged) dataset for model building. Nevertheless, the regression analyses summarized in S1 indicate that differences in gene expression level between whole blood and PBMC are sufficiently similar to make at least qualitative comparisons of predictive accuracies across these sample types and combinations thereof. To further address the contraints of comparison across sample types, the prediction model suggested in [8] was retrained using the dataset of the current study and evaluated using the same cross validation setup used for the evaluation of the network-central gene models. When gene sets identified in [7, 8] are used as model predictors with the merged training data, our network-based models perform favorably in comparison.

Another potentially significant caveat to the current study is the fact that due to limited data, the examined predictive models and biosignatures used only cross-validation analysis, as opposed to an independent external dataset for validation. As additional gene expression data in stroke patients become available, the prediction model based on network-central gene biosignatures should be subjected to further external validation with additional, independent data.

Apart from potentially achieving similar levels of predictive accuracy with a smaller array of genes, the principal advantage of the network-based approach lies in providing greater information content through the identification of genes with co-expression patterns correlated with those in the prediction model, i.e. the d = 1, 2 network neighbors. Network models leverage joint information about pairwise co-expression patterns, and so can provide additional insights into the functions and pathways of genes underlying a disease or condition. Such statistical associations generate hypotheses for future experimental confirmation of the significance of the identified network-central predictor genes and their neighbors to stroke physiology, and consequently, their potential to serve as biomarkers in clinical assays.

There are a number of possible biological explanations for the observed changes in network connectivity and gene centrality between stroke and control samples. The identification of 6 network-central genes whose expression levels are strongly characteristic of acute ischemic stroke is thought to be a consequence of specific, correlated patterns of gene expression linked to stroke and associated reactions to anoxia, inflammation, and cell death in both the brain and in blood vessels. Such correlated patterns of gene activity are particularly evident in the increased number of interactions (or at least statistically significant associations) between control and stroke for *NOG, BMX, SFXN2* and *SLC22A12* vs. the decrease in association with *ID3* and *MBTPS1,* as evidenced by the relative sizes of d = 1 neighborhoods.

While differential gene expression is necessary for the identification of gene centrality in a network, it is not necessarily the case that the most strongly dysregulated genes are associated with the overall biological changes driven by a particular disease or condition. The fact that none of the genes with the strongest differences in network centrality (with the exception of *ID3*) are characterized by with largest FC further illustrates the efficacy and power of network-based approaches to the analysis of gene expression. It is also noteworthy that significantly differentially expressed genes from independent studies such as *TTC7B* [4] were not found to be significant in the integrated dataset, either in the analyses of log FC differences or in the network models. This could indicate that the significant FC observed for this gene in individual studies are due to particular characteristics of that experimental design, or due to the gene expression outcomes specific to the therapy/drug treatments received by a specific cohort of patients.

A large difference in centrality score between stroke vs. non-stroke samples may be indicative of gene regulatory pathways that are disrupted (either differentially activated or repressed) during a stroke even in the absence of large changes in the expression levels of individual genes. For example, the existence of less highly connected neighborhoods associated with specific genes may provide insight into gene expression “modules” associated with specific pathways. This modularity is disrupted during stroke events (and potentially other pathological states) through the up or down-regulation of genes that link the once functionally and statistically separated pathways.

As a potential limitation to these interpretations, it is noted that some of the changes in mRNA density associated with stroke may be a “passive” result of cell death rather than due to stroke-specific changes in gene expression patterns. The genes associated with these mRNAs are less likely to be part of regulatory or signalling cascades unique to stroke, and as such of limited interest as possible therapeutic targets. This aspect is particularly relevant in the case of circulating miRNAs. miRNA/mRNA interactions occur in the cytoplasm, so changes in the concentrations of circulating miRNAs may be the result of differential expression of miRNA in cells that die and lyse.

Similar considerations apply to the differences in mRNA densities that are a consequence of changes in relative leukocyte densities rather than differential expression. Several recent papers [37–39] have shown that the density of lymphoid cells in whole blood decreases following stroke, while the relative abundance of neutrophils and other myeloid cells increases. Consequently, at least part of the changes to mRNA densities in whole blood reflect changes in relative leukocyte abundance rather than changes in transcription patterns as such.

Notably, the 10 genes used as stroke predictors in [7, 8] are differentially expressed between myeloid and lymphoid leukocytes, so that the change in relative cell count explains most of the observed variation [40]. Among these genes is *ID3*, which is also a predictor gene in the present study. However, whether differential expression vs. changes in leukocyte densities account for differential abundance of mRNA in the remaining 5 predictor genes and those in their network neighbors remains an open question. GO analyses of genes in the neighborhoods of the network-central predictors (and the predictor genes themselves, apart from *ID3*) do not indicate enrichment with respect to immune function or differentiation of blood cells. Furthermore, *ID3* is the only gene in the set of 6 which has among the highest differences in mRNA abundance between stroke and control, so it is possible that the patterns seen for the remaining 5 genes and their network neighbors are not being driven by the same processes as those leading to differential mRNA densities in *ID3*.

## Conclusions

The results of this study suggest a number of possible directions for future research. To further validate the applicability of the significant genes as biomarkers, it would be of value to repeat the analyses with a control set of stroke mimics (i.e. samples from non-AIS patients who exhibit stroke-like symptoms) rather than the healthy controls obtained from the GEO public data sets. Beyond the specific applicability of these results to distinguishing stroke from non-stroke, we can further refine our classification of clinical phenotypes according to patient outcomes (e.g. based on NIH Stroke Scale or Rankin scores) and associating these with characteristic gene expression patterns. This would allow us to identify biomarkers predictive of patient outcomes and perhaps eligibility for therapeutic interventions.

## Additional files

Additional file 1 : This file summarizes analyses comparing pooled whole blood + PBMC samples vs. whole blood only. **Figure S1.1** shows a volcano plot of FC vs. log *p*-values for the blood samples only, which is qualitatively consistent with Fig. 1 for pooled data. The second figure is a scatterplot and Spearman correlation analysis of pooled data FC vs. blood only FC, confirming consistency across the datasets. (DOCX 36 kb)

Additional file 2 : The file diff_exp_results_control_vs_stroke.txt provides a complete list of log FC and p-values for genes in stroke vs. control samples (TXT 1263 kb)

Additional file 3 : The file netcompbiomarkers_1395.txt lists the 47 genes identified as having statistically significant differences in centrality value between stroke and control (TXT 2 kb)

Additional file 4 : This file contains a complete list of Hamming distance d = 1 neighborhoods for the 6 predictive network-central genes, for both the control (non-stroke) and stroke gene expression networks. (TXT 9 kb)

Additional file 5 : DAVID enrichment clusters for the genes with statistically significant FC between stroke and control (**S5A**), as well as for Hamming distance d ≤ 1 neighborhoods for each of the network-central predictor genes: ***S5B*** – *ID3*, ***S5C*** – *MBTPS1*, ***S5D*** – *NOG*, ***S5E*** – *SFXN2*, ***S5F*** – *BMX*, ***S5G*** – *SLC22A1. (7Z 17 kb)*

Additional file 6 : Diseases associated with variants or differential expression of the 6 predictive network-central genes with a gene-disease association (GDA) or variant-disease association (VDA) score > 0.001 according to DisGeNet. (TXT 1 kb)

Additional file 7 : miRNA:mRNA target predictions – a complete list of statistically significant associations between log FC in miRNAs and mRNA log FC in the 6 network-central predictor genes, including the confidence scores. (TXT 29 kb)

## Data Availability

All data were obtained from the NIH GEO server and are thus publically available. The software used for most of the analyses is part of the proprietary InSyBio package and is available for purchase. The accession numbers and their respective reference publications are GSE22255 [4], GSE16561 [16], and GSE58294 [17].

## Terminology

AIS: Acute Ischemic Stroke
FS: Fold Change
GO: Gene Ontology
GEO: Gene Expression Omnibus
DAVID: Database for Annotation, Visualization, and Integrated Discovery
PCA: Principal Components Analysis
FDR: False Discovery Rate
KNN: K nearest neighbor
OR: Odds Ratio
LOF: Local Outlier Factor
GA: Genetic Algorithm
SVM: Support Vector Machine
mRNA: messenger RNA
miRNA: micro RNA
has-mir: human micro RNA
RNASeq: RNA Sequencing
qPCR: quantitative polymerase chain reaction.

## Genes

*ID3*: Inhibitor of DNA binding 3
*MBTPS1*: Membrane-bound transcription factor site-1 protease
*NOG*: Noggin
*SFXN2*: Sideroflexin-2
*BMX*: BMX non receptor tyrosine kinase
*SLC22A1*: Solute carrier family 22 member 1
*TTC7B*: Tetraricopeptide Repeat Domain 7B
*CAP3*: Cyclase Associated Protein 3
*NLK*: Nemo-Like Kinase
*ACTB*: Actin Beta
*B2M*: Beta-2 Microglobulin
*HMBS*: Hydroxymethylbilane Synthase
*HPRT1*: Hypoxanthine Phosphoribosyltransferase 1
*RPL13A*: Ribosomal Protein L13A
*SDHA*: Succinate Dehydrogenase Complex, Subunit A
*TBA*: Tubulin Alpha Chain
*YWHAZ*: Tyrosine 3-Monooxygenase/Tryptophan 5-Monooxygnease Activation Protein Zeta
*ALKBH2*: Alpha-Ketogularate dependent dioxygenase
*MFGE8*: Milk fat globule EGF factor 8/lactadherin
*LEF1*: Lymphoid enhancer binding factor 1
*TRIB2*: Tribbles pseudokinase 2
*FLT3LG*: Fms-related tyrosine kinase 3 ligand
