# Supplementary material for: Discovery of stroke-related blood biomarkers from gene expression network models
Source: BMC Med Genomics. 2019 Aug 7;12:118. doi: 10.1186/s12920-019-0566-8 (PMC6686563; doi:10.1186/s12920-019-0566-8)
Supplement: Supplementary file 1 — : This file summarizes analyses comparing pooled whole blood + PBMC samples vs. whole blood only. Figure S1.1 shows a volcano plot of FC vs. log p-values for the blood samples only, which is qualitatively consistent with Fig. 1 for pooled data. The second figure is a scatterplot and Spearman correlation analysis of pooled data FC vs. blood only FC, confirming consistency across the datasets. (DOCX 36 kb) [file 12920_2019_566_MOESM1_ESM.docx]

Supplementary Material 1

Among the datasets that have been combined in the present study there exist 40 samples which come from peripheral blood mononuclear cells. Statistical analysis has been conducted with and without these samples. The results of the analysis including these samples but correcting for the sample type (using the Ebayes method of limma package and stating the cell type as covariate variable) are depicted in Figure 1 of the manuscript. The results of the analysis without the PBMC samples are depicted in Supplementary Figure X.1 below:


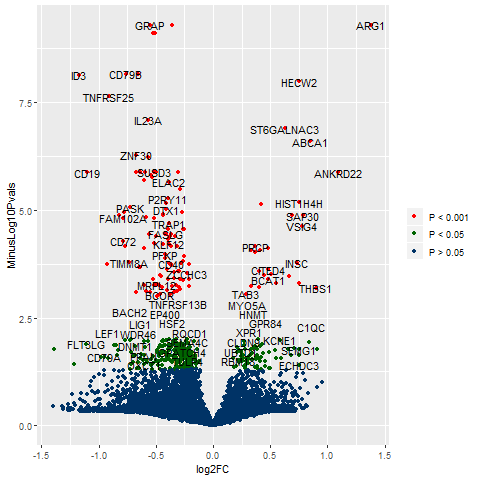


**Supplementary Figure S1.1:** Volcano plot of the comparison of control versus stroke samples without using the PBMC samples.

To further analyse the results of the statistical comparison using or excluding the PBMC samples, we performed Spearman correlation analysis comparing the log fold changes retrieved by both analysis. The results of this analysis are depicted in Supplementary Figure X.II and they show perfect correlation between the retrieved log fold changes. This finding enabled us to include the PBMC samples in our analysis.


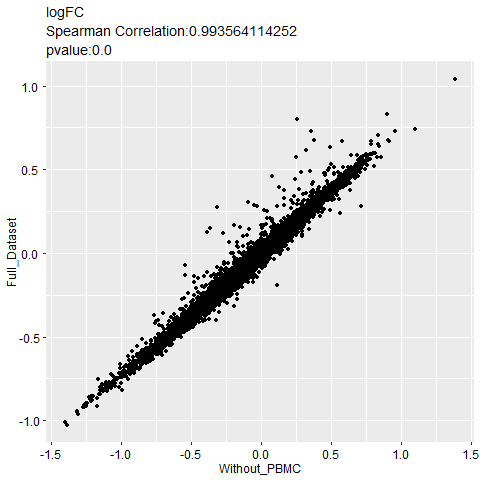


**Supplementary Figure S1.II** Spearman Correlation of the log_2­_ Fold Changes retrieved when using all samples and when excluding the PBMC samples.
